# Policy Implications for Local Application of the 2009 Youth Risk Behavior Survey, Duval County, Florida

**Published:** 2012-04-12

**Authors:** William C. Livingood, Thomas Bryant, Kathy Bowles, Dale Bell, Marcy LaVine, Rick Kane, Ryan Butterfield, Razaila Luminita, Rebecca Filipowicz

**Affiliations:** Duval County Health Department. Dr Livingood is also affiliated with the University of Florida College of Medicine, Jacksonville, Florida, and Georgia Southern University College of Public Health, Statesboro, Georgia; Duval County Health Department, Jacksonville, Florida; Duval County Public School Board, Jacksonville, Florida; Duval County Public School Board, Jacksonville, Florida; Duval County Public School Board, Jacksonville, Florida; Duval County Public School Board, Jacksonville, Florida; Duval County Public School Board, Jacksonville, Florida. Mr Butterfield is also affiliated with the University of Florida College of Medicine, Jacksonville, Florida, and Georgia Southern University College of Public Health, Statesboro, Georgia; Duval County Health Department, Jacksonville, Florida; Duval County Health Department, Jacksonville, Florida. Ms Filipowicz is also affiliated with the Texas Department of Health Services, Austin, Texas

## Abstract

**Introduction:**

Youth Risk Behavior Survey (YRBS) data have rarely been analyzed at the subcounty level. The purpose of this study was to explore the feasibility of such analysis and its potential to inform local policy and resource allocation.

**Methods:**

We administered the 2009 YRBS to 5,860 students from 46 public middle and high schools in Duval County, Florida. In addition to asking core questions, we asked a set of questions customized for local needs, including questions about zip codes. These data were used to simulate subcounty areas consistent with areas identified by behavioral, morbidity, mortality, and health disparity surveillance. We oversampled Duval County and used weighting procedures that adjusted for subcounty areas.

**Results:**

Many Duval County health risk behavior rates were higher than those for Florida overall but did not vary significantly within the county. Physical activity and violence-related behaviors were exceptions that reflect major health disparities in parts of the county with a high proportion of racial/ethnic minorities.

**Conclusion:**

This study demonstrated that collecting subcounty data in large metropolitan areas is feasible and that analysis of these data at the local level has implications for policy. Some health risk behaviors were common across the county, indicating the need for health promotion and disease prevention programs at the school district level. Other health risk behaviors were more prevalent in specific areas of the county and may have been exacerbated by state or local policies such as restrictions on physical education. Health disparities remain a challenge throughout the country; reducing them will require more extensive data-driven problem solving at state and local levels.

## Introduction

The Youth Risk Behavior Surveillance System (YRBSS), administered by the Centers for Disease Control and Prevention (CDC), monitors risky behaviors among children in the United States. State and local Youth Risk Behavior Survey (YRBS) data have been used to support policy development related to HIV and sexuality, physical fitness, and underage drinking in Florida, Texas, Mississippi, and Wyoming (1). YRBS data are reported nationally and for 42 states and 20 metropolitan areas.

Many of the leading causes of death for youth are behavioral. For example, in the United States, most deaths among youth and young adults result from motor-vehicle injuries, other unintentional injuries, homicide, and suicide (1). The leading causes of premature death for adults are also behavioral (tobacco use, lack of physical activity, and dietary behaviors); behavioral habits and preferences frequently are established in childhood. Other major health issues such as sexually transmitted diseases and immunization rates also have behavioral patterns frequently established in youth.

YRBS data have been used for policy analysis by comparing possible relationships between policy change and health-related behaviors. For example, secondary analysis of YRBS data was used to demonstrate that tobacco tax policies were associated with reduced tobacco use among youth (2). Analysis of surveillance data also has been used to assess the effect of mandatory seat belt laws on seat belt use, motor-vehicle fatalities, and crash-related injuries among youths (3); stricter enforcement of laws forbidding tobacco sales to minors (4); tobacco prices and taxes on youth tobacco use (5,6); and relationship of population density to youth alcohol use (7).

Behavioral surveillance has also been used to understand factors associated with unhealthy conditions and high-risk behaviors. For example, YRBS data for 1 condition or risk behavior is linked with data for other risk behaviors. Violence, substance abuse, sexual behaviors, obesity, suicidal behaviors, environmental exposure to tobacco, asthma, and insufficient sleep are some of the conditions or risk factors for which relationships have been studied using YRBS data (8-20).

We describe a way to analyze YRBS data at the subcounty level and explore the usefulness of this approach and the implications for community-level decision making.

## Methods

YRBS comprises a core set of standardized questions that have been tested for validity and reliability and are administered nationally (21). In addition to the core questions, customized questions can be included in the survey. The Duval County Public Schools, Duval County Health Department, YRBS Survey Advisory Team, and Jacksonville community stakeholders revised the YRBS instrument to fit the needs of the local community. In addition to questions to address specific topics, questions were added to facilitate subcounty analysis.

CDC procedures were followed for selecting the sample and for administering the survey (21). All 27 middle schools and 19 high schools in Duval County were included in the 2009 YRBS survey; classes were randomly selected.

Because zip code areas are statistically unreliable for analysis of many types of data, the Duval County Health Department had previously created 6 multiple–zip code health zones to provide reliable and consistent data for subcounty analysis. This spatial analytic technique is consistent with those used to assess behavioral, morbidity, mortality, and health disparity surveillance data in Duval County (22). The health zones have different demographic characteristics; 1 area is more than 80% African American and 2 others are less than 20% African American. Health zones also have major differences in social determinants, morbidity, and mortality. For example, health zone 1 has higher poverty rates, lower high school graduation rates, higher infant mortality, and higher diabetes mortality. Public and private agencies use the health zones for health and disease surveillance in Duval County.

Confidence intervals are the primary tool that CDC uses for comparing differences in YRBS responses (1,21), although the *t* test is also used to increase precision for assessing significance for bivariate comparisons (23). The responses were weighted according to CDC procedures (21,24) to increase precision for comparing frequencies of Duval County to Florida and among the 6 health zones. The *z* test for proportions (25) was also used to increase precision of significance testing for the comparison of Duval County to Florida and to confirm statistical significance for bivariate differences among the health zones for which differences were identified with confidence intervals. Significance was set at *P* < .05. (Information on statistical significance and weighting can be obtained from the corresponding author.)

## Results

**Figure 1.**
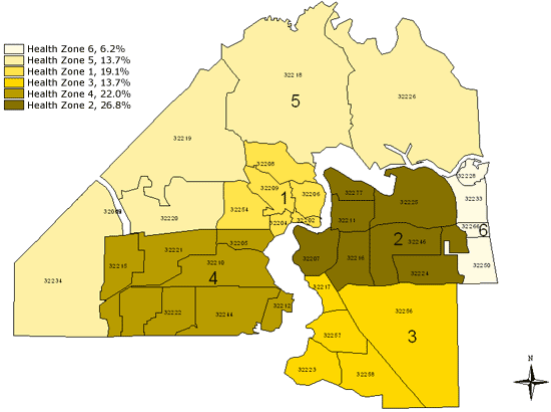
Map of health zones in Duval County, Florida, showing distribution of respondents to the 2009 Youth Risk Behavior Survey.

A total of 2,542 high school students from 19 public schools and 3,138 middle school students from 27 public schools participated in this survey. The proportionate responses ranged from 6.2% in health zone 6 to 26.8% in health zone 2 (Figure 1).

**Table:** 

**Health Zone 1, %**	**Health Zone 2, %**	**Health Zone 3, %**	**Health Zone 4, %**	**Health Zone 5, %**	**Health Zone 6, %**
19.1	26.8	13.7	22.0	13.7	6.2

### Middle school

**Violence, suicide, and safety behaviors**

Greater percentages of Duval County students reported violence-related behaviors compared to all Florida students (Table 1). A higher percentage of county middle school students carried weapons for protection overall and at school, had been in a fight at school, and had been bullied, compared with students statewide. Similarly, more county students considered suicide, made a suicide plan, or tried to commit suicide compared with students statewide. Nearly twice as many students in Duval County reported riding with a drinking driver as students statewide. Violence-related behaviors differed by health zone. The percentage of students in health zone 1 who carried a weapon to school for protection was almost double that of health zones 2, 3, and 6 (P < .001). Similarly, health zone 1 had the highest percentage of students who reported being in a fight at school. Safety behaviors varied across health zones; more than 92% of students in health zone 1 reported never or rarely wearing bicycle or skate helmets, higher than in all other health zones (P < .001).

**Alcohol and drugs**

We found numerous differences between the county and state for the use of tobacco, alcohol, and other drugs. Duval County students reported higher lifetime use of cigarettes, alcohol, marijuana, and inhalants. The rates for middle school students in health zone 1 who had ever tried cigarettes, alcohol, or marijuana were higher than for students in all the other health zones (P = .01). Rates for this category of behaviors did not vary by health zone.

**Physical activity**

**Figure 2.**
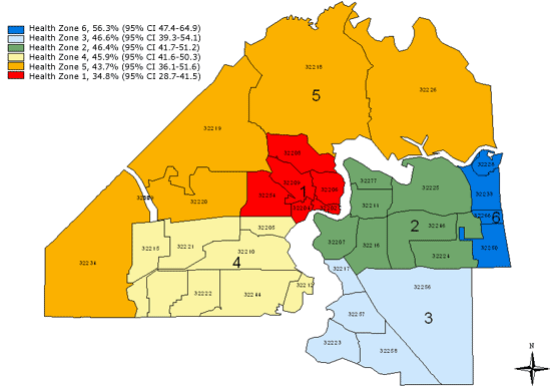
Map of health zones in Duval County, Florida, showing percentage of middle school students who were active at least 60 minutes on 5 or more days of the past 7 days, 2009 Youth Risk Behavior Survey.

**Figure 3.**
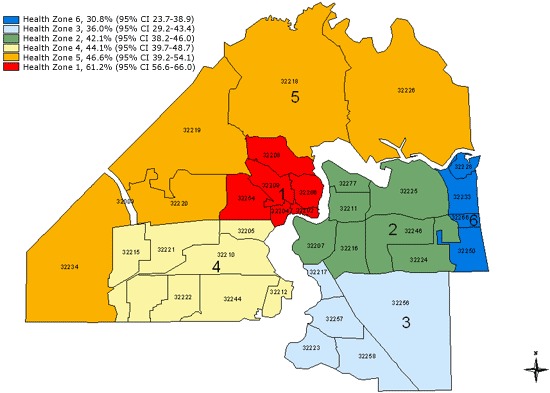
Map of health zones in Duval County, Florida, showing percentage of middle school students who watch an average of 3 or more hours of television daily, 2009 Youth Risk Behavior Survey.

Duval County middle school students, overall, were less active than students in Florida. A smaller percentage of Duval County students engaged in physical activity for at least 60 minutes on 5 or more days per week. Higher percentages of Duval County students also watched 3 or more hours of television daily and played video games 3 or more hours per day compared to students in Florida. However, a higher percentage of Duval County students attended physical education (PE) class at least 1 day per week than Florida students. The percentage of Duval County students who consumed 1 or more sodas on the previous day was higher than for Florida. There were no differences found among the health zones for most of these behaviors. However, health zone 6 had the most active students (56.3%) and health zone 1, the least active (34.8%) (P < .001) (Figure 2). Health zone 6 also had the highest percentage of students who attended PE classes daily, and health zone 5 had the highest rate of self-reported overweight students (26.3%) as well as the highest percentage of severe weight-loss behavior, including use of laxatives (10.4%) and vomiting (13.1%). Health zone 1 had the least desirable food consumption behaviors — only 11.0% of students reported having had 5 or more fruits and vegetables on the day previous to the survey — and had the highest percentage of students who, cumulatively, drank at least 1 soda, watched 3 or more hours of television daily (Figure 3), or played 3 or more hours of video games per day.

**Table:** 

Health Zone 1, % (95% CI)	Health Zone 2, % (95% CI)	Health Zone 3, % (95% CI)	Health Zone 4, % (95% CI)	Health Zone 5, % (95% CI)	Health Zone 6, % (95% CI)
34.8 (28.7-41.5)	46.4 (41.7-51.2)	46.6 (39.3-54.1)	45.9 (41.6-50.3)	43.7 (36.1-51.6)	56.3 (47.4-64.9)

**Table:** 

Health Zone 1, % (95% CI)	Health Zone 2, % (95% CI)	Health Zone 3, % (95% CI)	Health Zone 4, % (95% CI)	Health Zone 5, % (95% CI)	Health Zone 6, % (95% CI)
61.2 (56.6-66.0)	42.1 (38.2-46.0)	36.0 (29.2-43.4)	44.1 (39.7-48.7)	46.6 (39.2-54.1)	30.8 (23.7-38.9)

**Sexual behaviors**

A greater percentage of middle school students (35.5%) who resided in health zone 1 had engaged in sexual intercourse than students in all other health zones (range, 17.1%-21.9%; *P* < .001). Florida does not assess sexual behaviors among middle school students.

### High school

**Violence, suicide, and safety behaviors**

Twenty-three percent of Duval County high school students reported they had carried a weapon in the previous 30 days, significantly more than students statewide. The rate for Duval students who carried a weapon on school property in the 30 days before the survey was significantly higher than the rate for Florida. Duval County students also had significantly higher rates of being injured or threatened by a weapon in the previous 12 months. There were no differences for safety issues involving automobiles and drinking across the health zones, although a higher percentage of students in the county than the state said they rarely or never wear a seat belt. Responses were similar across the health zones.

**Alcohol and drugs**

Rates of lifetime methamphetamine use and lifetime inhalant use for the county was higher than for the state. The percentage of Duval County students offered, sold, or given an illegal drug on school property in the past 12 months was higher than that for Florida. Comparison of data across the health zones revealed few differences, but students in health zone 4 had the highest percentage of current cigarette use (16.8%), while health zone 1 had the lowest percentage (6.8%) (*P* < .001). Seventy percent of high school students in health zone 5 had ever consumed alcohol, compared to 60% of students in health zone 1 (*P* = .004).

**Physical activity**

Duval County high school students had less sufficient physical activity than Florida students as a whole, and the proportion of students who did not attend PE classes daily in Duval County was higher than for Florida. The number of high school students who ate less than 5 servings of fruits and vegetables was higher for Duval County than for the state. The percentage of students in health zone 1 who reported watching television 3 or more hours per day (57%) was higher than for health zones 2, 3, and 6, each with less than 39% (*P* < .001). The percentage of obesity was highest among students in health zone 4 (17%) and lowest among those in health zone 1 (9%) (P = .001). In addition, 35% of high school students in health zone 5 drank at least 1 can or bottle of soda per day in the previous 7 days, compared to 26% in health zone 2 (P = .003).

**Sexual behaviors**

Of Duval County students who were currently sexually active, 58% used condoms, compared to 65% of state students. Duval County students had less education about HIV/AIDS than Florida students as a whole. Fourteen percent of students in health zone 1 had sexual intercourse for the first time when they were younger than 13, a significant difference from health zones 2, 3, and 4 (*P* < .01). The percentage of students currently sexually active in health zone 1 was also higher than for students in health zones 3, 4, and 5 (*P* = .003).

## Discussion

This was one of the first attempts to use YRBS to conduct subcounty analysis. We observed few differences among the subcounty health zones of Duval County, despite the differing demographic characteristics of these zones. Duval County's youth had a higher prevalence of risky behaviors than the rest of Florida's youth. Both similarities and differences at the subcounty level have policy implications; in some cases, districtwide policies are needed, and in other cases, more specific policies and resource allocation are needed for specific parts of the county. When a risk is significant throughout the county, identifying, monitoring, and communicating its severity is the first step in creating the community awareness that is necessary to mobilize community resources to address it.

However, some risk behaviors were more prevalent in some parts of the county. For example, the percentage of middle school students who reported fighting and carrying a weapon was highest in health zone 1, the urban core. Middle school students in the urban core also reported less physical activity, more sedentary behaviors, and poorer nutrition behaviors. Disparities between middle school students living in the urban core and those in the other health zones may be linked to other health disparities in health zone 1, such as high rates of diabetes deaths, hospitalization, and emergency department use (25). The lack of opportunity for physical education in conjunction with the lack of physical activity in the urban core has implications for state and local education policies, which have tended to reduce physical activity. On the other hand, students in other zones were more likely to engage in tobacco or alcohol use. Identifying and monitoring where these problems are most prevalent can allow policy makers to direct resources appropriately. For example, increased physical education and opportunities for physical activity may be a higher priority for parts of the community where disparities exist. In particular, the review and revision of policies that decrease opportunities for physical activity as punishment or increase time for remedial education should be considered.

Our analysis of YRBS data in the middle school population showed the importance of middle school behavioral surveillance. Florida does not collect data on sexual activity for middle school students, and our county data showed that middle school students were engaging in high-risk sexual behaviors. Another surprising result from YRBS was the high percentage of Duval County middle school students who used tobacco. It is unclear whether this finding is an anomaly or represents a reversal of the accomplishment in reducing youth tobacco use a decade earlier. This high rate of tobacco use needs to be monitored at the middle school level to determine whether the prevalence of tobacco use increases as these middle school students advance to high school; such a trend may support reinstituting tobacco control policies that were weakened early in the decade.

Middle school YRBS is conducted much less frequently across the country than high school YRBS. Tobacco use also illustrates how middle school YRBS can be a valuable tool to assess the effect of policy. Changes in tobacco policies and practices related to youth tobacco use appeared to reduce youth tobacco use a decade ago. Although many of those policies and the funding for those practices have been discontinued, youth tobacco use has remained consistent. Davis et al (26) found that cuts in the Florida Tobacco Control Program budget resulted in significant declines in exposure to some interventions, particularly tobacco countermarketing. Monitoring the effect of these reductions in exposure to tobacco countermarketing efforts may be necessary to determine whether there is a residual effect of the tobacco control program, and the residual effect is wearing off.

Physical activity is another risk behavior that may be influenced by policies. In Florida, students whose performance on state assessments in reading or math is low are required to enroll in a remedial course, which may waive their physical education requirement. This policy may have a greater effect on racial/ethnic minority students in Duval County, who tend to lag in academic progress and have much lower graduation rates (27). The lower rates of regular physical activity and high rates of television viewing and video gaming in the high-minority urban core (health zone 1) indicate that these students are at highest risk of the detrimental effects of low physical activity, and these are the students most likely to be deprived of organized physical activity during the school day. This finding has policy implications related to school support for physical activity, given the high rates of death, hospitalization, and emergency department use for health conditions linked to sedentary behaviors in the same area (health zone 1) of the city (22). Where behavioral risk is documented to be more persistent, resources and policies such as school policies that affect physical activity, can be adjusted to address those problems. Analysis at the subcounty level may be useful.

Previously identified limitations of YRBS apply to this study; all YRBS data are self-reported, students who were absent during survey administration were unaccounted for, and the extent of underreporting or overreporting of behaviors cannot be determined, although YRBSS data are consistently of acceptable quality (21). Limitations specific to this study include the smaller sample sizes for 2 of the health zones, which reduced the reliability of some results of the subcounty analysis. These lower sample sizes also decreased the number of health zone comparisons that could have been significant.

This study documented the feasibility of customizing the YRBS to enable analysis of behavioral risk factors at the subcounty level. Such an analysis can inform policy decisions and resource allocation on the basis of which high-risk behaviors are common to subcounty health zones and which are concentrated in specific zones. Many of these behaviors can be addressed through school- or community-based interventions.

## Figures and Tables

**Table 1.** Characteristics of Middle School Students in Duval County, Florida (n = 3,188), Compared to All Florida Middle School Students (N = 6,356), 2009 Youth Risk Behavior Survey

**Risk Factor**	**Duval County Students, % (95% CI)**	**Florida Students, % (95% CI)**	***P* Value^a^**
**Violence**			
Carried weapon for protection	32.2 (30.3-34.4)	18.1 (16.4-19.9)	<.001
Carried weapon for protection at school	9.8 (8.6-11.3)	2.3 (1.9-2.7)	<.001
Been in a fight at school	46.3 (44.2-48.7)	18.5 (16.9-20.1)	<.001
Been bullied at school	33.3 (31.3-35.3)	28.6 (27.0-30.3)	<.001
Been electronically bullied	17.6 (16.1-19.5)	21.3 (20.0-22.6)	<.001
**Suicide**			
Thought about suicide	21.5 (19.9-23.2)	15.2 (14.2-16.1)	<.001
Made suicide plan	14.5 (13.3-16.2)	8.5 (7.8-9.3)	<.001
Attempted suicide	10.3 (9.1-11.6)	5.6 (5.0-6.2)	<.001
**Safety**			
Never/rarely wore bicycle helmet	83.3 (81.4-85.2)	79.9 (77.9-81.9)	<.001
Never/rarely wore skate helmet	83.6 (81.2-86.0)	85.2 (83.4-87.1)	.04
Never/rarely wore seat belt	13.4 (12.0-15.2)	10.8 (9.5-12.0)	<.001
Rode with drinking driver	35.1 (33.1-37.2)	18.8 (17.6-20.0)	<.001
**Tobacco**			
Ever tried cigarettes	29.4 (27.2-31.9)	19.1 (17.4-20.8)	<.001
Smoked first cigarette before age 11	6.4 (5.3-7.7)	DNA	NA
Smoked ≥1 cigarette in past 30 d	7.9 (6.8-9.6)	6.5 (5.6-7.4)	.01
**Alcohol**			
Drink alcohol ever	43.8 (40.6-47.1)	31.9 (30.1-33.6)	<.001
Had first drink before age 11	18.5 (16.8-20.3)	13.3 (12.2-14.4)	<.001
**Other drug use**			
Ever use marijuana	17.9 (16.0-19.9)	10.9 (9.9-11.9)	<.001
Ever use inhalants	16.1 (14.4-17.9)	10.2 (9.4-11.1)	<.001
**Physical activity**			
Active ≥60 min on ≥5 of the past 7 d	44.4 (42.0-46.9)	49.2 (46.9-51.6)	<.001
Watched ≥3 h/d of television	44.6 (42.2-47.0)	40.8 (38.3-43.2)	<.001
Played video games ≥3 h/d	30.8 (29.0-32.7)	28.7 (27.1-30.3)	.03
Attended PE class ≥1 d/wk	79.6 (77.0-81.9)	60.6 (56.6-64.7)	<.001
Played on ≥1 sports team in past 12 mo	55.1 (52.9-57.4)	72.8 (71.1-74.5)	<.001
Attended PE class daily	41.0 (38.3-43.5)	44.2 (38.6-49.7)	.003
**Obesity, body image, and dietary behaviors**			
Slightly/very overweight (self-reported)	24.7 (22.9-26.5)	25.3 (24.0-26.6)	.53
Took diet pills to lose weight	7.1 (6.2-8.2)	3.7 (3.1-4.3)	<.001
Vomited/took laxatives to lose weight	7.8 (6.8-8.9)	4.2 (3.5-4.8)	<.001
Ate ≥5 fruits and/or vegetables yesterday	11.7 (10.4-13.1)	DNA	NA
Drank soda ≥1 time yesterday	55.2 (53.1-57.3)	28.7 (26.9-30.5)	<.001
Had energy drink ≥1 time yesterday	22.5 (20.4-24.9)	DNA	NA
**Sexual behavior**			
Ever had sexual intercourse	23.3 (21.1-25.7)	DNA	NA
Had sexual intercourse for the first time at age <11 y	8.2 (7.0-9.6)	DNA	NA
Had sexual intercourse with >3 people during lifetime	7.9 (6.6-9.3)	DNA	NA
Used a condom during last sexual intercourse	66.8 (62.4-70.9)	DNA	NA

Abbreviations: CI, confidence interval; DNA, did not ask; NA, not applicable; PE, physical education.

a Calculated by using the *z* test of proportions.

**Table 2.** Characteristics of High School Students in Duval County, Florida (n = 2,542), Compared to all Florida High School Students (N = 5,684), 2009 Youth Risk Behavior Survey

**Risk Factor**	**Duval County Students, % (95% CI)**	**Florida Students, % (95% CI)**	***P* Value^a^**
**Violence**			
Carried weapon for protection	22.3 (20.3-24.6)	17.3 (16.1-18.5)	<.001
Carried weapon on school property	7.8 (6.6-9.3)	4.7 (4.0-5.5)	<.001
Did not go to school because they felt unsafe at/to/from school	14.4 (12.8-16.1)	6.9 (6.1-7.8)	<.001
Threatened or injured with a weapon on school property	13.9 (12.0-16.0)	8.2 (7.4-9.0)	<.001
In a physical fight	34.9 (32.4-37.6)	29.8 (28.1-31.5)	<.001
**Suicide**			
Seriously considered attempting suicide	14.2 (12.8-15.8)	11.6 (10.7-12.7)	.01
Attempted suicide	9.9 (8.4-11.8)	6.5 (5.8-7.4)	<.001
**Safety**			
Never/rarely wore seat belt	16.1 (14.2-18.0)	11.6 (10.3-13.0)	<.001
Rode with drinking driver	30.8 (28.7-33.1)	27.6 (26.1-29.2)	.02
Drove a vehicle after drinking	11.1 (9.1-13.6)	DNA	NA
**Tobacco**			
Ever tried cigarettes	28.4 (26.1-30.7)	DNA	NA
Current cigarette use	15.4 (13.4-17.7)	16.1 (14.8-17.5)	.58
Current smokeless tobacco use	9.2 (7.4-11.3)	7.1 (6.1-8.2)	.06
**Alcohol**			
Drank alcohol ever	66.0 (63.5-68.4)	DNA	NA
Current alcohol use	38.8 (35.9-41.9)	40.5 (38.5-42.6)	.35
Episodic heavy drinking	19.7 (17.4-22.2)	21.1 (19.6-22.8)	.32
**Other drug use**			
Ever use marijuana	38.6 (35.9-41.5)	36.4 (34.7-38.1)	.18
Ever use cocaine	7.4 (6.0-9.1)	6.9 (6.2-7.7)	.57
Ever use methamphetamine	6.9 (5.5-8.6)	4.2 (3.5-5.1)	<.001
Ever use inhalants	14.3 (12.4-16.5)	10.8 (9.5-12.3)	<.001
Offered, sold, or given an illegal drug on school property	36.8 (34.3-39.4)	21.8 (20.4-23.3)	<.001
**Physical activity**			
Active ≥60 min on ≥5 of the past 7 d	30.1 (28.0-32.3)	40.8 (39.1-42.4)	<.001
Did not attend PE classes daily	91.6 (90.2-92.9)	73.3 (70.3-76.0)	<.001
Watched ≥3 h/d of television	41.0 (38.3-43.5)	38.2 (36.3-40.1)	.09
**Obesity, body image and dietary behaviors**			
Obese (calculated BMI based on self-reported height, weight)	12.9 (11.4-14.7)	10.3 (9.3-11.4)	.01
Ate fruits and vegetables <5 times/d	81.8 (79.7-83.7)	78.4 (77.1-79.7)	.01
Drank soda ≥1 time/d in last 7 d	29.6 (27.5-31.3)	28.6 (26.9-30.4)	.46
Vomited/took laxatives to lose weight or keep from gaining weight	8.6 (7.1-10.4)	4.5 (3.9-5.1)	<.001
**Other health-related risk factors**			
Lifetime asthma	24.5 (22.3-26.9)	20.7 (19.6-21.8)	<.001
**Sexual behavior**			
Ever had sexual intercourse	53.9 (50.5-57.2)	50.6 (48.1-53.1)	.12
Had sexual intercourse for the first time before age 13 y	10.1 (8.5-12.1)	8.3 (7.3-9.3)	.07
Had sexual intercourse with 4 or more people during lifetime	17.6 (15.4-20.0)	16.6 (15.4-17.8)	.41
Currently sexually active	37.7 (34.6-40.8)	37.0 (34.8-39.3)	.72
Used a condom during last sexual intercourse	58.3 (54.1-62.6)	65.1 (63.0-67.1)	.01
Ever been taught in school about HIV/AIDS	84.3 (82.2-86.2)	88.2 (86.4-89.7)	<.001

Abbreviations: CI, confidence interval; DNA, did not ask; NA, not applicable; PE, physical education; BMI, body mass index.

a Calculated by using the *z* test of proportions.
